# Sources of Vitality of the Teeth

**Published:** 1853-07

**Authors:** 


### SOURCES OF VITALITY OF THE TEETH.

This is now a subject of considerable interest, and perhaps more noticed from the introduction of the new operation, called by two or three appellations besides the gentleman's names who introduces it. Risodontropy, Rhizodentrypo, Neurhæemaxis, &c. Can the vitality of the nerve exist after separation at the point of the fang, and when the tooth has no union with the alveolus or gum except on its palital half?

We have a case to the point, which we commend to the writer of "source of vitality in the teeth," June 22d, 1853, in the Boston Medical and Surgical Journal. One of our oldest and most reputable surgeons in the West or United States, gives us the following: A lower incisor in his own mouth had become loosened from absorption of the gum and Alveolus, and the point of the fang was uncovered, he removed this tooth—split it open and found the pulp alive. We know this case staggers belief. Yet we are sure that Dr. M. is worthy of all credence, and is withal a close and accurate observer, and not apt to be deceived in such investigations. We would ask the writer in the Boston Medical and Surgical Journal, if the cementum petrosa and periosteum are the same—or is this portion of the root supplied from the periosteum with nourishment?

